# Efficient Plant Regeneration and Transient Genetic Transformation System of *Prunus xueluoensis* via an *Agrobacterium*-Mediated Method

**DOI:** 10.3390/ijms26083588

**Published:** 2025-04-10

**Authors:** Yang-Yang Lin, Shui-Han Wu, Jie Chen, Xian-Gui Yi, Xian-Rong Wang, Meng Li

**Affiliations:** Co-Innovation Center for Sustainable Forestry in Southern China, College of Life Sciences, Nanjing Forestry University, Nanjing 210037, China; lin-yangyang@foxmail.com (Y.-Y.L.); w1277480685@163.com (S.-H.W.); chenjie@njfu.edu.cn (J.C.); yixiangui@njfu.edu.cn (X.-G.Y.); wangxianrong66@njfu.edu.cn (X.-R.W.)

**Keywords:** *Prunus xueluoensis*, regeneration system, transient expression, transient genetic transformation, *Agrobacterium*-mediated

## Abstract

*Prunus xueluoensis*, a unique *Prunus* germplasm resource native to China, exhibits significant ornamental value due to its short juvenile phase, early flowering period, abundant flowers, and elegant tree form. However, the lack of an efficient regeneration and genetic transformation system has hindered its genetic improvement and wider application. In this study, we focused on optimizing the tissue culture conditions for *P. xueluoensis* and establishing an *Agrobacterium*-mediated transient genetic transformation system. We first determined the optimal medium compositions for different stages of tissue culture, including seed germination, callus induction, adventitious bud differentiation, and rooting. For seed germination, the optimal medium was MS supplemented with 200 mg/L GA3 and 4 mg/L 6-BA. For callus induction, the best medium was MS containing 2.00 mg/L 6-BA, 1.00 mg/L NAA, and 200 mg/L VC. Adventitious bud differentiation was favored on MS medium with 1.00 mg/L 6-BA, 0.10 mg/L NAA, and 200 mg/L VC, while rooting was optimal on 3/4 MS medium supplemented with 0.50 mg/L NAA. Subsequently, we established an *Agrobacterium*-mediated transient genetic transformation system using stem segments of *P. xueluoensis* as explants. Through orthogonal experiments, we identified the optimal conditions for genetic transformation as pre-cultivation for 2 days, an *Agrobacterium* concentration of OD_600_ = 0.6, an infection time of 30 min, and co-cultivation for 3 days. Under these conditions, the transient genetic transformation efficiency reached 10.42%, as confirmed by PCR and *GFP* fluorescence detection. This study provides a reliable transient genetic transformation system for *P. xueluoensis*, facilitating further functional gene analysis and genetic improvement of this valuable ornamental species.

## 1. Introduction

Introduced by Nan and colleagues in 2013 [1], *Prunus xueluoensis* represents a novel species within the Prunus genus, distinguished by its unique characteristics. It is distributed in China [2] and is considered a significant genetic resource within cherry plants of the Rosaceae family. Like other ornamental species, such as the Chinese jujube (*Ziziphus jujuba*) [3] and sweet cherry (*Prunus avium*) [4], which are known for their short juvenile phase and early flowering, *Prunus xueluoensis* is postulated to exhibit similar characteristics, including a short juvenile phase, early flowering, abundant flowers, elegant coloration, and a low-growing habit. These traits make it valuable for genetic research due to its unique characteristics and rapid flowering and fruiting. These traits would make it an excellent material for bonsai cultivation and an attractive option for genetic research and breeding programs aimed at creating novel ornamental varieties. The genus *Prunus* encompasses approximately 150 species with over 500 varieties, exhibiting considerable genetic diversity [5]. Among them, *P. xueluoensis* stands out due to its extensive natural distribution and the availability of numerous cultivated varieties. Despite its ornamental potential, the natural distribution of *P. xueluoensis* is limited to specific mountainous regions at altitudes of 1100 to 1500 m. Additionally, its seed germination rate under natural conditions is low, which is further complicated by the inefficiency of its regeneration system and the lack of an established genetic transformation protocol. These factors have hindered the advancement of molecular studies and restricted the application and popularization of this species. Therefore, there is an urgent need to establish efficient tissue culture and genetic transformation systems for *P. xueluoensis*. Such advancements would greatly support efforts in resource conservation, germplasm innovation, and molecular breeding programs. Previous studies have already made significant strides in understanding the tissue culture and genetic transformation of various *Prunus* species, including *Prunus cerasifera* [6], *Prunus avium* [7], *P. persica* [8], and *P. mume* [9]. These investigations have provided invaluable insights into the factors that influence regeneration and transformation efficiency within the *Prunus* genus.

Tissue culture techniques offer a promising avenue for the rapid propagation and conservation of rare and endangered plant species [10]. By employing plant growth regulators and optimized media compositions, researchers can induce callus formation, adventitious [11] shoot regeneration, and rooting, thereby establishing in vitro regeneration systems [12]. Previous studies have successfully established tissue culture protocols for various *Prunus* species, demonstrating the feasibility of this approach for *P. xueluoensis* as well [13,14,15]. Genetic transformation, particularly through *Agrobacterium*-mediated methods, has emerged as a powerful tool for introducing desirable traits into plant genomes [16,17]. This technique leverages the natural ability of *Agrobacterium tumefaciens* to transfer DNA into plant cells, offering advantages such as high efficiency, low cost, and clear transfer fragments. By integrating foreign genes into the plant genome, researchers can confer resistance to biotic and abiotic stresses, enhance growth characteristics, and improve the overall value of the crop [18]. Despite the potential benefits, the genetic transformation of woody plants, including *P. xueluoensis*, remains challenging due to factors such as genotype sensitivity, explant type, and the complex interaction between *Agrobacterium* and plant cells. Previous studies on genetic transformation in *Prunus* species have primarily focused on herbaceous model plants or economically important fruit crops, with limited research on ornamental tree species like *P. xueluoensis*. Therefore, there is a need to systematically investigate the key factors affecting the efficiency of *Agrobacterium*-mediated genetic transformation in *P. xueluoensis* and to establish an optimized protocol suitable for this species.

To the best of our knowledge, the establishment of an efficient genetic transformation system for *P. xueluoensis* remains understudied, with most research focusing on its living environment, seed characteristics, phenotypic and genetic variation, gene cloning, and leaf regeneration systems [1,19]. Currently, challenges such as the selection of suitable explants, low regeneration rates, and difficulties in disinfecting explant surfaces hinder methodological advancements. Notably, there have been no prior reports on the successful development of a genetic transformation system for *P. xueluoensis* using stem segments as explants [20]. In the present study, we utilized stem segments from aseptic seedlings of *P. xueluoensis* as explants and established a highly efficient and stable regeneration system by optimizing the types and concentrations of plant growth regulators, as well as culture conditions. We also explored the feasibility of *Agrobacterium*-mediated genetic transformation using the green fluorescent protein (*GFP*) gene as a reporter. By systematically investigating various factors influencing transformation efficiency, including the optical density of *Agrobacterium* [21,22] tumefaciens (OD_600_), infection time [23], the duration of pre-cultivation and co-cultivation [24], and the concentration of the selective agent [25], this study provides a reliable regeneration and transient genetic transformation system for *P. xueluoensis*, thereby facilitating genetic improvement for breeding and laying the groundwork for the creation of varieties with superior market potential for this valuable ornamental species.

## 2. Results

### 2.1. Development of an Efficient In Vitro Regeneration System for Prunus xueluoensis

#### 2.1.1. Effects of Disinfection Methods on Seed Germination

**Figure 1:**
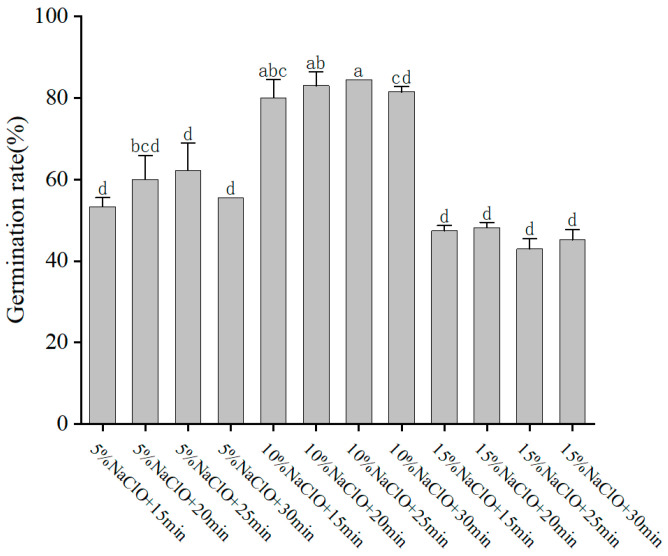
Effects of disinfection methods on seed germination in *Prunus xueluoensis*. The highest germination rate of 84.44% was achieved with 75% alcohol for 5 min, followed by 10% NaClO for 25 min. Different lowercase letters above bars indicate significant differences (*p* < 0.05) according to Duncan’s multiple range test. Abbreviations: NaClO—Sodium hypochlorite.

To establish an efficient in vitro regeneration system for *P. xueluoensis*, the first step was to determine the optimal disinfection method for seeds. Twelve different disinfection treatments, varying in NaClO concentration and duration, were tested. Figure 1 indicates that a combination of 75% alcohol for 5 min, followed by 10% NaClO for 25 min, yielded the highest germination rate of 84.44%. This treatment was significantly better than the others, as evidenced by statistical analysis. This finding suggests that a proper balance of disinfection strength and duration is crucial for maintaining high germination rates while minimizing damage to the seeds.

#### 2.1.2. Optimization of Medium Composition for Embryo Germination

**Figure 2:**
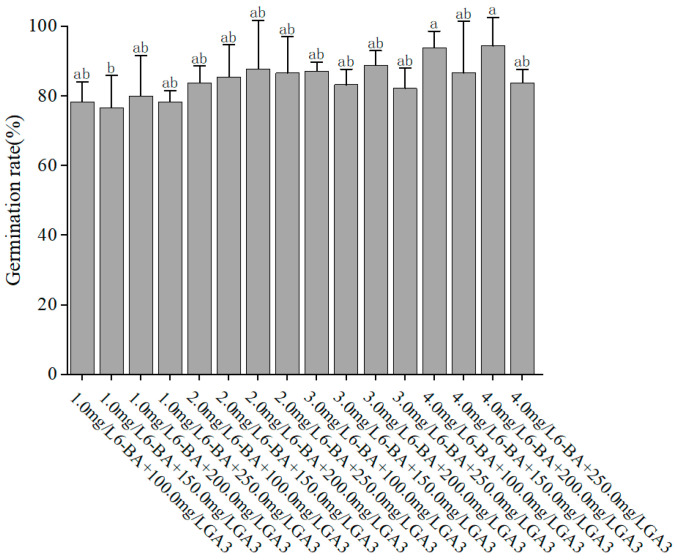
Effect of different PGRs on seeds germination in *Prunus xueluoensis*. Different lowercase letters above bars indicate significant differences (*p* < 0.05) according to Duncan’s multiple range test.

To optimize the medium for embryo germination, different combinations of 6-BA and GA3 were tested. As presented in Figure 2, the medium supplemented with 4 mg/L 6-BA and 200 mg/L GA3 yielded the highest germination rate of 94.44%. The ANOVA results indicated that the concentration of 6-BA had a significant effect on germination (F = 4.015, *p* = 0.016), with 4 mg/L being optimal. This suggests that a suitable balance of cytokinin and gibberellic acid is essential for promoting embryo germination in *P. xueluoensis*.

#### 2.1.3. Induction of Callus and Adventitious Buds

**Figure 3:**
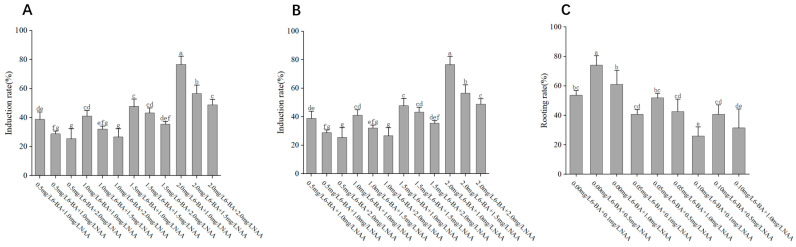
(**A**) Effect of different PGRs on callus in *Prunus xueluoensis*; (**B**) Effect of different PGRs on adventitious buds in *P. xueluoensis*; (**C**) Effect of different PGRs on roots in *P. xueluoensis*. Different lowercase letters above bars indicate significant differences (*p* < 0.05) according to Duncan’s multiple range test.

**Figure 4:**
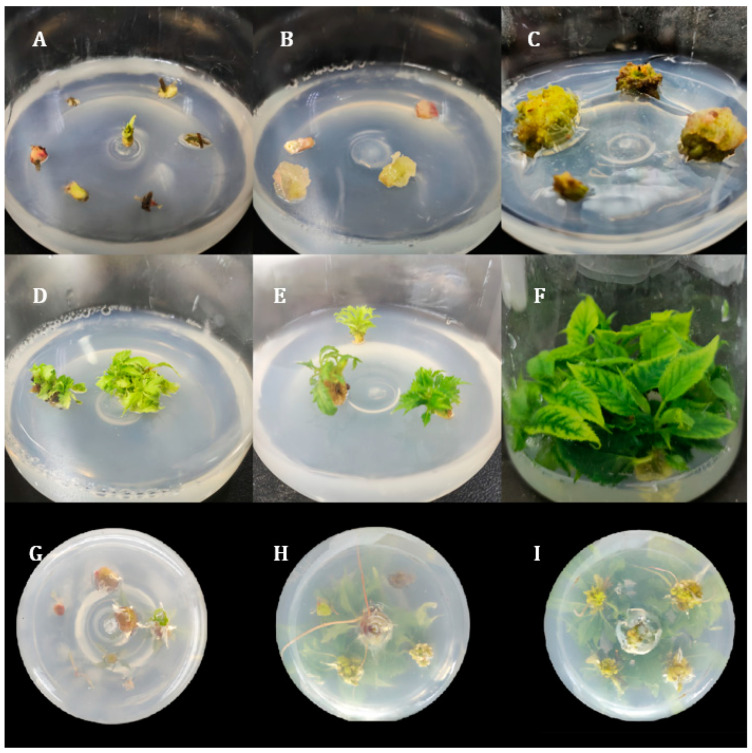
(**A**–**C**) Callus culture of stems with axillary buds on MS medium supplemented with 2.00 mg/L 6-BA, 1.00 mg/L NAA, and 200 mg/L VC. (**D**–**F**) Differentiation culture of adventitious buds from callus on MS medium containing 1.00 mg/L 6-BA, 0.10 mg/L NAA, and 200 mg/L VC. (**G**–**I**) Rooting culture of adventitious shoots with terminal buds on 3/4 MS medium supplemented with 0.50 mg/L NAA.

The initiation and growth of callus were significantly influenced by the combination and concentration of plant growth regulators (PGRs) (Figure 1 and Figure 2). As shown in Figure 3A and Figure 4, the optimal medium for callus induction was MS supplemented with 2.00 mg/L 6-BA, 1.00 mg/L NAA, and 200 mg/L VC. This combination yielded the highest callus induction rate of 76.66%, which was significantly higher than that of all other treatments (*p* < 0.05).

The differentiation of adventitious buds from callus was highly dependent on the concentrations of cytokinins and auxins in the medium (Figure 3 and Figure 4). As illustrated in Figure 3B and Figure 4, the combination of 1.00 mg/L 6-BA and 0.10 mg/L NAA in MS medium supplemented with 200 mg/L VC yielded the highest adventitious bud differentiation rate of 75.92%. Notably, as the concentration of NAA increased, the differentiation rate decreased, suggesting that low auxin levels are favorable for bud formation. This finding underscores the delicate balance required between cytokinins and auxins for optimal bud differentiation.

#### 2.1.4. Rooting of Adventitious Buds

**Figure 5:**
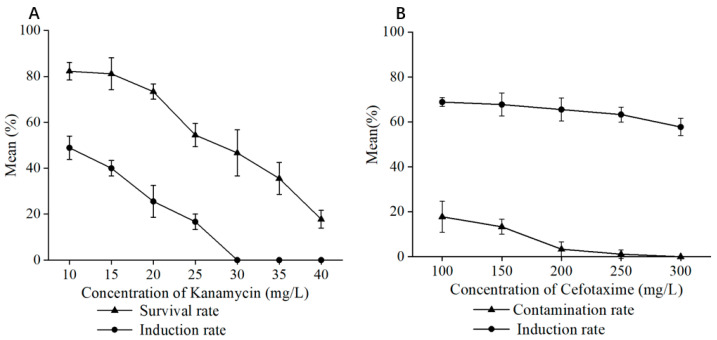
(**A**) Adventitious bud induction rate and survival rate at different Kanamycin concentrations; (**B**) Adventitious bud induction rate and contamination rate at different Cefotaxime concentrations.

**Figure 6:**
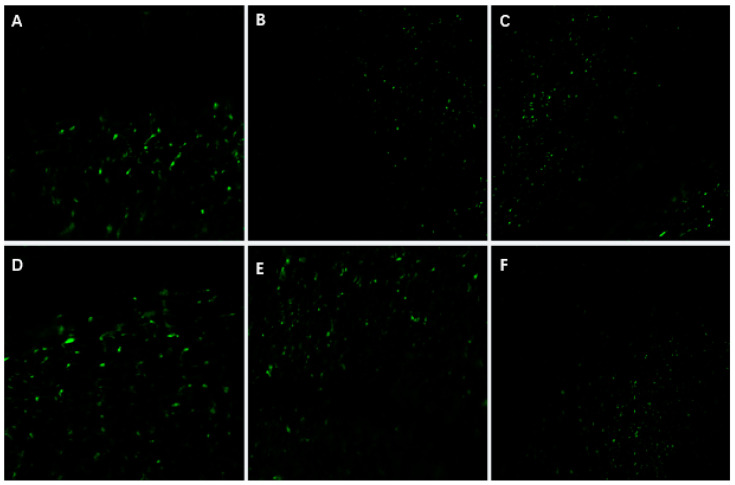
*GFP* fluorescence detection in leaves from putative transgenic *P. xueluoensis*. Photos (**A**–**F**) represent different transgenic lines showing punctate distribution of *GFP* fluorescence (green, 488 nm excitation). No *GFP* signals were observed in negative controls. Magnification: 20×. Abbreviations: *GFP*—Green Fluorescent Protein.

To explore the optimal conditions for rooting adventitious buds, we conducted experiments with various concentrations of 6-BA while keeping the concentration of NAA constant at 0.50 mg/L across different medium formulations (Figure 5 and Figure 6). Our results demonstrated that, when the concentration of NAA was kept constant, the highest rooting efficiency was achieved with a zero concentration of 6-BA, indicating that the absence of 6-BA is most favorable for adventitious bud rooting. Conversely, when the concentration of 6-BA was held constant, the highest induction rate was observed at an NAA concentration of 0.50 mg/L, suggesting that this concentration is optimal for adventitious bud rooting.

Based on a comprehensive analysis, the optimal medium formulation for rooting adventitious buds of *P. xueluoensis* was determined to be 3/4 MS supplemented with 0.50 mg/L NAA, achieving an average rooting rate of 74.08% (Figure 3C). This finding indicates that the combination of reduced inorganic salt strength in the medium (3/4 MS) and an appropriate concentration of auxin (NAA) is crucial for stimulating root development in adventitious buds of *P. xueluoensis*. Statistical analysis revealed a significant effect of NAA concentration on rooting performance, underscoring the importance of fine-tuning auxin levels to optimize root induction. Specifically, the rooting rate increased with increasing NAA concentration up to 0.50 mg/L, after which further increases did not yield proportionate benefits and, in some cases, led to a slight decline. This suggests that while auxin is essential for root initiation, excessive concentrations may interfere with the rooting process.

### 2.2. Establishment of an Agrobacterium-Mediated Genetic Transformation System

#### 2.2.1. Sensitivity of Explants to Antibiotics

To determine the appropriate antibiotic concentrations for selection, untransformed leaf explants were cultured in medium containing different concentrations of kanamycin (Kan) and cefotaxime (Cef) (Figure 5). The results indicated that 30 mg/L Kan effectively inhibited the growth of non-transformed tissues without affecting the growth of transformed cells. For Cef, a concentration of 250 mg/L was found to adequately suppress *Agrobacterium* growth while causing minimal damage to the explants.

#### 2.2.2. Optimization of *Agrobacterium*-Mediated Transformation Conditions

An orthogonal experimental design was employed to optimize pre-cultivation time, *Agrobacterium* concentration (OD_600_), infection time, and co-cultivation time. The results (Table 1) showed that the factors influencing transformation efficiency, in descending order, are as follows: *Agrobacterium* concentration > pre-cultivation time > infection time > co-cultivation time. This suggests that the bacterial solution concentration has the greatest impact on conversion efficiency. Based on the average transformation efficiency corresponding to each level of the factors, the highest values are found at level two of factor A, level two of factor B, level three of factor C, and level one of factor D. Thus, the optimal combination is determined to be A2B2C3D1, which corresponds to a pre-culture time of 2 days, a bacterial solution concentration of OD_600_ = 0.6, an infection time of 30 min, and a co-culture time of 3 days. Under these conditions, the genetic transformation efficiency of *P. xueluoensis* reaches up to 10.42%.

##### Influence of Pre-Culture Time on Transformation Efficiency

Pre-culturing the explants prior to *Agrobacterium* infection enhanced their susceptibility to genetic transformation. Three pre-culture durations (0 d, 2 d, and 4 d) were evaluated. The results indicated that a pre-culture period of 2 days resulted in the highest transformation, likely due to the optimal physiological state of the explants for T-DNA integration (Table 2).

##### Effect of Bacterial Solution Concentration on Transformation

The concentration of the *Agrobacterium* suspension significantly impacted transformation efficiency. Bacterial solutions with OD_600_ values of 0.4, 0.6, and 0.8 were tested. The results demonstrated that an OD_600_ of 0.6 yielded the highest transformation rate, suggesting that this concentration optimized the balance between sufficient T-DNA delivery and minimal explant damage (Table 3).

##### Impact of Infection Time on Transformation

The duration of *Agrobacterium* infection was critical for effective genetic transformation. Infection times of 10 min, 20 min, and 30 min were compared. The results showed that an infection time of 30 min resulted in the highest transformation efficiency, indicating that this duration allowed sufficient time for T-DNA transfer without causing excessive explant stress (Table 4).

##### Effect of Co-Culture Time on Transformation

The co-culture period following infection allowed for T-DNA integration into the plant genome. Co-culture times of 3 days, 4 days, and 5 days were evaluated. The results indicated that a co-culture duration of 3 days was optimal, as longer co-culture periods led to increased *Agrobacterium* overgrowth and explant necrosis, thereby reducing transformation efficiency (Table 5).

#### 2.2.3. Molecular and Phenotypic Confirmation of Transgenic Plants

##### *GFP* Fluorescence Detection

Using fluorescence microscopy, *GFP* gene expression can be observed as green fluorescence under specific blue excitation light wavelengths. In this experiment, leaves from healthy *P. xueluoensis* plants, selected after screening, were used for fluorescence microscopy (Figure 6). The results showed that green fluorescence was detected in the leaf samples, which appeared as punctate distributions rather than continuous patches, indicating that some cells were successfully infected and that the *GFP* gene was introduced.

##### PCR Analysis of Transgenic Plants

**Figure 7:**
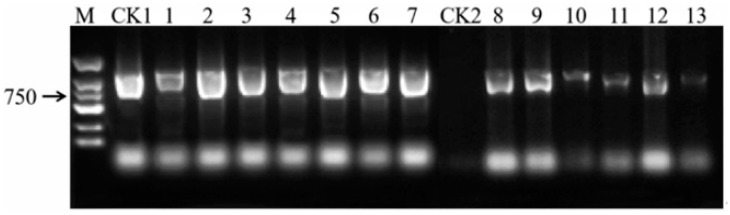
PCR-validated electrophoresis of transgenic plants. M: 2000 DNA marker; CK1: Positive control; CK2: Negative control; 1–13: Transgenic plants. The amplification of the expected GFP gene fragment confirms the genetic modification in the transformed plants. Abbreviations: PCR—Polymerase Chain Reaction.

PCR analysis using *GFP*-specific primers was conducted to further verify the presence of the transgene in putative transgenic plants. The results (Figure 7) demonstrated the amplification of the expected *GFP* gene fragment in five independent transgenic lines, confirming their genetic modification. Genetic transformation efficiency was determined by counting the number of PCR-positive and *GFP*-fluorescent plants and expressing this as a percentage of the total number of explants used.

## 3. Discussion

In this study, the efficient in vitro regeneration and *Agrobacterium*-mediated transient genetic transformation systems for *P. xueluoensis* were systematically investigated. The results obtained provide valuable insights into the genetic engineering of this endangered species and offer a solid foundation for future functional genomics studies.

### 3.1. Effect of Plant Growth Regulators on Tissue Culture

The selection and concentration of plant growth regulators (PGRs) are crucial for successful tissue culture and plant regeneration. In our study, the combination of MS medium supplemented with 200 mg/L GA3 and 4 mg/L 6-BA was found to be optimal for seedling germination, achieving a high germination rate of 94.44%. During initial experiments on disinfection methods, we found that the germination rate of embryos reached up to 84.44% when grown on medium without auxins. Upon introducing NAA at specific concentrations, we noted variations in the germination patterns. Notably, the inclusion of 1.00 mg/L NAA suggested a possible enhancement in germination, although this improvement did not reach statistical significance. These preliminary findings underscore the need for further investigation to conclusively determine the role of auxin in promoting embryo germination, as the current data suggest a trend rather than a definitive conclusion. These results are consistent with previous studies indicating that Nan Chenghui [1] found that adding GA3 and 6-BA to the germination culture of *P. discoidea* could better break dormancy and promote sprouting. Similarly, the medium with MS and a combination of 2.00 mg/L 6-BA, 1.00 mg/L NAA, and 200 mg/L VC was most effective for callus induction, yielding a callus induction rate of 76.66%. This indicates that cytokinin is a key factor in callus induction, while auxin plays a secondary role. These results are consistent with previous studies indicating that an appropriate balance between cytokinins and auxins is essential for callus formation and shoot regeneration in woody plants [26,27].

For adventitious shoot differentiation, the medium containing MS with 1.00 mg/L 6-BA, 0.10 mg/L NAA, and 200 mg/L VC promoted the highest differentiation rate of 75.92%. In contrast, the rooting medium composed of 3/4 MS and 0.50 mg/L NAA was optimal for rooting, achieving a rooting rate of 74.08%. These findings suggest that lower concentrations of auxins favor root development, while higher concentrations promote shoot formation, which aligns with previous reports on cherry tissue culture [28].

### 3.2. Antibiotic Concentration Screening and Bacteriostatic Concentration Screening

In the process of establishing the genetic transformation system for *P. xueluoensis*, screening suitable concentrations of antibiotics and bacteriostatic agents is crucial for the successful selection of transgenic plants and the prevention of contamination caused by *Agrobacterium*. The present study systematically investigated the effects of different concentrations of kanamycin (Kan) and cefotaxime (Cef) on the growth and transformation efficiency of *P. xueluoensis* explants. Kanamycin, commonly used as a selective agent in plant genetic transformation, effectively inhibits the growth of non-transgenic tissues while allowing transgenic plants to thrive. Our results revealed that a Kan concentration of 30 mg/L was optimal for screening transformed *P. xueluoensis* stem segments, as it effectively suppressed the growth of non-transformed tissues without adversely affecting the transformed explants. This concentration is comparable to those reported for other woody plants, although it may vary depending on the species and explant type [29]. For instance, studies on the genetic transformation of lilies have demonstrated the utility of antibiotics like hygromycin, kanamycin, and glyphosate for resistance screening [25]. It is important to note that the selection of the appropriate Kan concentration must balance effectively eliminating non-transformed tissues and minimizing the negative impact on transformed explants [30].

In addition to selective antibiotics, bacteriostatic antibiotics play a crucial role in genetic transformation by preventing post-infection contamination of the receptor material by *Agrobacterium* and mitigating the inhibitory effects of *Agrobacterium* growth on the transformation process, thereby reducing the likelihood of recipient cell death and regeneration difficulties. Cephalosporins, known for their broad-spectrum antibacterial activity and relatively low toxicity to plant cells, are commonly employed as bacteriostatic agents in plant genetic transformation. Furthermore, this study revealed that the concentration of the bacteriostatic agent is intertwined with the concentration of *Agrobacterium* used for infection. Specifically, at lower *Agrobacterium* concentrations, minimal or no bacterial colonies were observed, suggesting an interplay between the two that warrants careful consideration during experimental design.

### 3.3. Factors Influencing Genetic Transformation Efficiency

The efficiency of *Agrobacterium*-mediated genetic transformation is influenced by various factors, including pre-culture time, *Agrobacterium* concentration, infection time, and co-culture time. In our study, the optimal conditions for transforming *P. xueluoensis* were determined to be a pre-culture time of 2 days, an *Agrobacterium* concentration of OD_600_ = 0.6, an infection time of 30 min, and a co-culture time of 3 days. Under these conditions, the genetic transformation efficiency reached a maximum of 10.42%, as determined by PCR and *GFP* fluorescence detection. This efficiency is comparable to that of *Prunus cerasifera* [6] but lower than that of herbaceous models [17], reflecting inherent challenges in woody plant transformation.

Pre-culture has been shown to enhance the susceptibility of explants to *Agrobacterium* infection by priming the cells for transformation [25]. Our study determined that a 2-day pre-culture period is optimal, as it enables cells to enter a phase of active division, thereby increasing their receptivity to T-DNA transfer. This duration likely primes explant cells for T-DNA uptake by enhancing mitotic activity, as evidenced in *Prunus avium* [4].

The concentration of *Agrobacterium* plays a critical role in transformation efficiency. An OD_600_ value of 0.6 was found to be optimal, as higher concentrations could cause excessive damage to the explants, leading to reduced transformation efficiency. This finding is consistent with previous studies that report that moderate *Agrobacterium* concentrations are essential for successful transformation [31].

Infection time is another important parameter affecting transformation efficiency. An infection period of 30 min was determined to be optimal in our study [32]. Shorter infection times may not allow sufficient time for T-DNA transfer, while longer times can cause excessive damage to the explants [33].

Co-culture time also significantly impacts transformation success. A co-culture period of 3 days was found to be ideal, as it allowed sufficient time for T-DNA integration into the plant genome without causing excessive *Agrobacterium* growth and subsequent explant damage. These findings are in agreement with previous reports emphasizing the balance between T-DNA transfer and explant health during co-culture [34]. It is important to note that the genetic transformation achieved in this study is transient, meaning that the introduced T-DNA is not stably integrated into the plant genome. The 3 days of co-cultivation were found to be sufficient for T-DNA delivery and initial expression of the *GFP* gene, as evidenced by *GFP* fluorescence detection. However, further studies are needed to achieve stable genetic transformation in *Prunus xueluoensis*.

### 3.4. Implications and Future Directions

To contextualize the efficiency of our regeneration and transient transformation system, we compared our results with previously reported studies on other *Prunus* species (Table 6). For instance, regeneration efficiencies in *Prunus cerasifera* [6] (76.66% callus induction) and *P. avium* [7] (65–70% shoot regeneration) align closely with our findings for *P. xueluoensis* (76.66% callus induction, 75.92% adventitious bud differentiation). However, the transient transformation efficiency in our study (10.42%) exceeds the values reported for *P. mume* [9] (6.5%) and *P. persica* [8] (3–5%), likely due to optimized *Agrobacterium* infection and co-cultivation conditions. Notably, the transformation of *P. xueluoensis* using stem segments as explants represents a methodological advancement, as earlier studies predominantly used cotyledons or leaves. These comparisons underscore the robustness of our protocol and its applicability to understudied ornamental species within the *Prunus* genus.

The establishment of an efficient regeneration and transient genetic transformation system for *P. xueluoensis* has significant implications. First, it enables the rapid propagation of this rare species through tissue culture, aiding conservation efforts. Second, it provides a foundation for genetic improvement, such as introducing stress resistance or enhancing ornamental traits. The punctate *GFP* fluorescence observed in leaves (Figure 6), validated by PCR amplification of the *GFP* gene (Figure 7), confirms transient expression, which is consistent with prior studies in *Prunus* species [6,9]. While southern blot analysis remains essential for verifying stable integration, our focus here is on transient transformation, which does not require genomic integration for validation.

The transient nature of this system is evident in the localized *GFP* signals, which reflect T-DNA expression in individual cells rather than stable genome integration. Future studies will prioritize stable transformation by optimizing selection markers, regeneration media, and alternative methods (e.g., protoplast transformation). Additionally, advanced imaging techniques (e.g., confocal microscopy with cellular markers) will resolve the subcellular localization of *GFP* signals, addressing current limitations in spatial resolution.

## 4. Materials and Methods

### 4.1. Plant Materials and Culture Conditions

#### Optimization of Disinfection Methods on Seed Germination

High-quality seeds of *P. xueluoensis* were collected from a cultivated orchard in Nanjing, China (119°16′43″ E, 31°45′17″ N). Prior to cultivation, the seeds were processed by removing the fleshy outer layer and soaking them in sterile water at an initial temperature of 45 °C for 4 h. To determine the optimal disinfection protocol for the explants, twelve different combinations of sodium hypochlorite (NaClO) concentrations and immersion times were tested. The seeds were first rinsed with sterile distilled water and then treated with 75% ethanol for 5 min to remove surface contaminants. Subsequently, the seeds were disinfected with NaClO solutions at concentrations of 5%, 10%, and 15% for 15, 20, 25, and 30 min, respectively. Following NaClO treatment, the seeds were rinsed three times with sterile distilled water for 5 min each to remove any residual disinfectant. Each treatment included 45 seeds, and the experiments were replicated three times. Following sterilization, seeds were sown on Murashige and Skoog (MS) medium supplemented with 3% sucrose and solidified with 0.8% agar. The pH of the medium was adjusted to 5.8 before autoclaving at 121 °C for 20 min. Seeds were cultured in a growth chamber maintained at 25 ± 2 °C under a 16 h photoperiod with a light intensity of 40 μmol m^−2^ s^−1^. The germination rate was assessed after 14 days by counting the number of seeds that had emerged radicles.

### 4.2. Plant Regeneration

#### 4.2.1. Optimization of Embryo Germination Medium

To determine the optimal medium for embryo germination, different combinations of plant hormones were tested. Sterilized seeds were sown on MS medium supplemented with varying concentrations of 6-benzylaminopurine (6-BA; 1, 2, 3, and 4 mg/L) and gibberellic acid (GA3; 100, 150, 200, and 250 mg/L). Each treatment was replicated three times, with 60 seeds per replicate. Germination rates were recorded after 25 days of culture.

#### 4.2.2. Callus and Adventitious Shoots Induction

Sterilized seeds were germinated on MS medium for 30 days, and aseptic seedlings were used as the source of explants. Explants consisting of axillary bud-bearing stem segments were transferred to callus induction medium containing MS salts supplemented with different concentrations of 6-benzylaminopurine (6-BA; 0.5, 1.0, 1.5, and 2.0 mg/L), naphthalene acetic acid (NAA; 1.0, 1.5, and 2.0 mg/L), and ascorbic acid (VC; 200 mg/L). The use of axillary bud-bearing stem segments as explants is a common practice in tissue culture and genetic transformation studies of woody plants, as these explants are known to have high regenerative capacity and transformation efficiency. The media were adjusted to pH 5.8 and solidified with 0.8% agar. Each treatment was replicated three times, with 30 explants per replicate. After 20 days of culture, a subset of calluses from each replicate of each callus induction treatment was randomly selected and transferred to shoot differentiation medium containing MS salts supplemented with 6-BA (0.5, 1.0, and 1.5 mg/L), NAA (0.05, 0.10, and 0.15 mg/L), and VC (200 mg/L). Each treatment was replicated three times, with 18 explants per replicate. After 30 days of culture, the number of explants producing adventitious shoots was recorded, and shoot induction rates were calculated.

#### 4.2.3. Adventitious Bud Differentiation

Adventitious buds induced from stem segments were transferred to MS medium containing different concentrations of 6-BA (0.5, 1.0, 1.5 mg/L) and NAA (0.05, 0.10, 0.15 mg/L) for elongation. After 30 days, the height of the elongated shoots was recorded, and the optimal medium for shoot elongation was identified.

#### 4.2.4. Root Induction and Plantlet Acclimatization

Adventitious buds, which were robustly growing and reached a height of 3 to 4 cm, were subjected to root induction culture. Using 3/4MS as the basic medium, these adventitious buds were inoculated onto rooting media containing various concentration combinations of 6-BA (0.00, 0.05, and 0.10 mg/L) and NAA (0.10, 0.50, and 1.00 mg/L). Root growth was periodically observed, and after 40 days, the rooting percentage of the adventitious buds was recorded. Once the roots reached 3–5 cm in length, plantlets were transferred to plastic pots containing sterile nutrient soil and vermiculite in a 2:1 ratio and placed in a greenhouse with controlled environmental conditions for acclimatization.

### 4.3. Agrobacterium-Mediated Genetic Transformation

#### 4.3.1. Plasmid and *Agrobacterium* Strain

**Figure 8:**
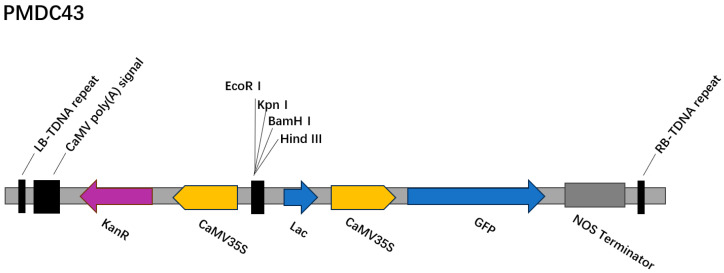
Linear (partial) restriction map of the *PMDC43* vector.

The plasmid used in this study was *PMDC43* [35] (Figure 8), which carries the *GFP* gene and a kanamycin (Kan) resistance marker. The plasmid was obtained from a commercial vector collection and has been previously used in plant genetic transformation studies. The plasmid was transformed into competent cells of *Agrobacterium tumefaciens* strain GV3101 (Novagen, Vadodara, India, Catalog No. 638976) [36] using chemical transformation. Single colonies were selected and verified by PCR amplification. Verified colonies were cultured in liquid Luria-Bertani (LB) medium containing 50 mg/L kanamycin and 50 mg/L rifampicin at 28 °C and 150 rpm overnight.

#### 4.3.2. Antibiotic Sensitivity of Explants

To determine the optimal concentrations of kanamycin (Kan) and cefotaxime (Cef) for the selection and inhibition of *Agrobacterium*, respectively, a series of concentrations were tested. Untransformed leaf explants were cultured in the optimal medium supplemented with kanamycin (Kan) (10, 15, 20, 25, 30, 35, and 40 mg/L). The leaf explants infected with *Agrobacterium* were individually cultured with cefotaxime (Cef) (100, 150, 200, 250, and 300 mg/L). The growth and survival of the explants were observed after 4 weeks of culture. The lowest concentration of Kan that effectively inhibited the growth of non-transformed tissues without affecting the growth of transformed cells was selected. Similarly, the lowest concentration of Cef that adequately suppressed *Agrobacterium* growth while causing minimal damage to the explants was determined.

#### 4.3.3. Pre-Culture and *Agrobacterium* Infection

For adventitious shoot induction, callus tissues induced from axillary bud-bearing stem segments of sterile seedlings were used as explants for co-cultivation with *Agrobacterium*. The efficiency of genetic transformation in *P. xueluoensis* was evaluated by examining the impact of various factors, including pre-cultivation time, *Agrobacterium* concentration (OD_600_), infection duration, and co-cultivation duration. Each treatment was replicated three times, with a total of 405 explants used in this study. The explants, consisting of stem segments approximately 1 cm in length and bearing axillary buds, were sourced from sterile seedlings of *P. xueluoensis* and were cultured on MS medium supplemented with 2.00 mg/L 6-BA, 1.00 mg/L NAA, and 200 mg/L VC for different pre-cultivation periods of 0, 2, and 4 days prior to infection. *Agrobacterium* strain GV3101 carrying the *PMDC43* construct was used for infection, with OD_600_ values set at 0.4, 0.6, and 0.8 to determine the optimal bacterial concentration. Infection durations of 10, 20, and 30 min were tested to assess their influence on transformation efficiency (Figure 7). Following infection, the explants were co-cultivated on the same medium used for pre-cultivation, but with the addition of 100 μmol/L acetosyringone (AS) to promote *Agrobacterium* infection for 3, 4, and 5 days to observe the effect of co-cultivation time on transformation. Cultures were maintained under a photoperiod of 14–16 h of light per day, with light intensity ranging from 1500 to 3000 Lux, at a temperature of 25 ± 1 °C.

### 4.4. Molecular Analysis of Transformed Plants

#### 4.4.1. DNA Extraction and PCR Analysis

Genomic DNA was extracted from the leaves of putative transformants after they had been grown on selection medium for several weeks, as well as from untransformed control plants, using a polysaccharide and polyphenol plant total DNA extraction kit (Catalog No. DP360, Tiangen Biotech Co., Ltd., Beijing, China). The presence of the exogenous *GFP* gene in the transformed plants was confirmed by PCR amplification. Specific primers for the *GFP* gene (Forward: 5′-GCGTGCAACTCGCTGATCATT-3′; Reverse: 5′-TAATCATCGCAAGACCGGCA-3′) were used. PCR reactions were performed at a total volume of 20 μL, containing 30–40 ng of genomic DNA, 10 μL of 2× Taq Master Mix (Tiangen, Beijing, China), 2 μL of each primer, with a final concentration of each primer of 1 μM in the PCR reaction mixture (35S-F: 5′-CAATCCCACTATCCTTCGCAAGACC-3′, Nos-R: 5′-TAATCATCGCAAGACCGGCA-3′), and distilled water to achieve the final volume. The PCR cycling conditions were as follows: initial denaturation at 94 °C for 3 min, followed by 35 cycles of denaturation at 94 °C for 30 s, annealing at 58 °C for 30 s, and extension at 72 °C for 30 s, with a final extension at 72 °C for 5 min. The PCR products were separated on a 1.5% agarose gel and visualized using a gel imaging system (Maigaode, Nanjing, China).

#### 4.4.2. Detection of *GFP* Fluorescence

*GFP* fluorescence detection was performed to evaluate the expression of the *GFP* gene in transformed *P. xueluoensis* plants infected with *Agrobacterium* at different stages. Leaf tissues from both transformed and non-transformed plants (serving as negative controls) were collected and placed on a glass slide for observation. A biological microscope (ML31) equipped with the MShot Image Analysis System version 1.1.6 was utilized for this purpose. Under the microscope, *GFP* signals were captured using a 488 nm excitation laser and a 500–550 nm emission filter to minimize interference from chlorophyll autofluorescence. Non-transgenic control plants exhibited no detectable *GFP* fluorescence under identical imaging conditions, confirming the specificity of the observed signals. Spectral separation was prioritized by avoiding overlap with chlorophyll autofluorescence (typically excited at 640 nm and emitted in the 660–720 nm range), ensuring that *GFP*-specific signals were distinguishable from background autofluorescence.

### 4.5. Statistical Analysis

The data were analyzed using SPSS 24.0 software. A one-way analysis of variance (ANOVA) was used to determine significant differences between treatments, and Duncan’s multiple range test was employed for post hoc comparisons. Statistical significance was set at *p* < 0.05.

## 5. Conclusions

In this study, an efficient regeneration and *Agrobacterium*-mediated transient genetic transformation system was successfully established for *P. xueluoensis*, a rare and valuable ornamental cherry species endemic to China. By optimizing the combination of PGRs, the most effective medium for callus induction and adventitious bud regeneration from seed embryos was identified as MS supplemented with 200 mg/L GA3 and 4 mg/L 6-BA. Furthermore, the regeneration system was employed to develop a genetic transformation protocol using the *PMDC43* vector harboring the *GFP* reporter gene. Key factors influencing transformation efficiency, including pre-cultivation time, *Agrobacterium* concentration, infection duration, and co-cultivation period, were systematically investigated. The optimal transformation conditions were determined to be a pre-cultivation time of 2 days, an *Agrobacterium* concentration of OD_600_ = 0.6, an infection duration of 30 min, and a co-cultivation period of 3 days. Under these conditions, a transformation efficiency of 10.42% was achieved, as confirmed by both *GFP* fluorescence detection and PCR analysis. This study not only provides a reliable protocol for the regeneration and genetic manipulation of *P. xueluoensis* but also lays a solid foundation for future genetic improvement and molecular breeding of this unique cherry species. While the current study focused on establishing a transient genetic transformation system, future research will explore methods for achieving stable integration of T-DNA, and we will focus on optimizing conditions for stable transformation, including southern blot analysis to confirm genomic integration, to advance the genetic improvement of *P. xueluoensis*, which is essential for creating varieties with superior market potential.

## Figures and Tables

**Table 1:** Range analysis of factors for orthogonal experiment.

Treatment	A ^a^	B	C	D	No. of Explants	No. of Transformed Plants	Transformation Rate
1	1	1	1	1	48	1	2.08
2	1	2	2	2	48	1	2.08
3	1	3	3	3	48	0	0
4	2	1	2	3	48	2	4.17
5	2	2	3	1	48	5	10.42
6	2	3	1	2	48	1	2.08
7	3	1	3	2	48	2	4.17
8	3	2	1	3	48	1	2.08
9	3	3	2	1	48	0	0
K1	4.16	10.42	6.24	12.5			
K2	16.67	14.58	6.25	8.33			
K3	6.25	2.08	14.59	6.25			
K1 mean value	1.387	3.473	2.08	4.167			
K2 mean value	5.557	4.86	2.083	2.777			
K3 mean value	2.083	0.693	4.863	2.083			
R	3.474	4.167	2.783	2.084			

^a^ Factors A, B, C, and D represent pre-cultivation time (days), *Agrobacterium* concentration (OD_600_), infection time (minutes), and co-cultivation time (days), respectively. Levels 1, 2, and 3 correspond to the specific values assigned to each factor in the orthogonal experimental design.

**Table 2:** Effects of pre-cultured day on transformation of *Prunus xueluoensis*.

Pre-Cultured Day (d)	Transformed Shoot Frequency (%)
0	1.37 ± 1.85b ^a^
2	5.88 ± 0.00a
4	2.46 ± 1.85b

^a^ Values represent the mean (±SE) of three independent experiments. Means followed by the same letter in the same column are not significantly different from each other at *p* ≤ 0.05 level according to Duncan’s multiple range test.

**Table 3:** Effects of OD_600_ values of *A. tumefaciens* liquid on transformation of *Prunus xueluoensis*.

OD_600_ Values	Transformed Shoots Frequency (%)
0.4	3.38 ± 1.85a ^a^
0.6	5.27 ± 0.00b
0.8	0.68 ± 1.85a

^a^ Values represent the mean (±SE) of three independent experiments. Means followed by the same letter in the same column are not significantly different from each other at *p* ≤ 0.05 level according to Duncan’s multiple range test.

**Table 4:** Effects of infection time on transformation of *Prunus xueluoensis*.

Infection Time (min)	Transformed Shoots Frequency (%)
10	2.22 ± 1.85a ^a^
20	2.68 ± 0.00b
30	4.98 ± 1.85a

^a^ Values represent the mean (±SE) of three independent experiments. Means followed by the same letter in the same column are not significantly different from each other at *p* ≤ 0.05 level according to Duncan’s multiple range test.

**Table 5:** Effects of co-culture time on transformation of *Prunus xueluoensis*.

Co-Culture Time (d)	Transformed Shoots Frequency (%)
3	4.32 ± 1.85a ^a^
4	3.89 ± 1.85b
5	2.02 ± 1.85a

^a^ Values represent the mean (±SE) of three independent experiments. Means followed by the same letter in the same column are not significantly different from each other at *p* ≤ 0.05 level according to Duncan’s multiple range test.

**Table 6:** Comparison of regeneration and genetic transformation efficiencies in *Prunus* species.

Species	Explant Type	Regeneration Efficiency (%)	Transformation Efficiency (%)	Key Methodology Differences	Reference
*Prunus xueluoensis*	Stem segments	76.66 (callus), 75.92 (buds)	10.42 (transient)	Stem explants; OD_600_ = 0.6, 30-min infection	Current study
*Prunus cerasifera*	Cotyledons	76.66 (callus)	/	Cotyledon explants	[6]
*Prunus mume*	Immature cotyledons	65.0 (somatic embryos)	6.5 (stable)	Cotyledon-derived somatic embryos	[9]
*Prunus persica*	Meristematic Bulks, MBs	70.0 (shoots)	3.0–5.0 (stable)	High auxin/cytokinin ratio	[8]
*Prunus avium*	Axillary buds	65–70 (shoots)	/	TIBA supplementation	[7]

## Data Availability

All data are available within the article.

## Abbreviations

The following abbreviations are used in this manuscript:

MS (Culture medium)	Murashige–Skoog
NAA (Auxin analog)	Naphthylacetic acid
6-BA (Cytokinin)	6-Benzylaminopurine
AS (Chemical material)	Acetosyringone
GA3 (Gibberellin)	Gibberellic acid
VC (Ascorbic acid)	Ascorbic acid
Kan (Antibiotic)	Kanamycin
Cef (Antibiotic)	Cefotaxime
GFP	Green Fluorescent Protein
